# Engagement With Digital Adherence Technologies as Measures of Intervention Fidelity Among Adults With Drug-Susceptible Tuberculosis and Health Care Providers: Descriptive Analysis Using Data From Cluster-Randomized Trials in Five Countries

**DOI:** 10.2196/62881

**Published:** 2025-07-28

**Authors:** Jason Alacapa, Amare Worku Tadesse, Natasha Deyanova, Tanyaradzwa Dube, Andrew Mganga, Rachel Powers, Job van Rest, Norma Madden, Egwuma Efo, Salome Charalambous, Kristian van Kalmthout, Degu Jerene, Katherine Fielding

**Affiliations:** 1 KNCV Tuberculosis Plus, Manila, Philippines; 2 TB Centre and Department of Infectious Disease Epidemiology and International Health, London School of Hygiene & Tropical Medicine, Keppel St, London, WC1E 7HT, United Kingdom, 44 20 7636 8636, 44 20 7636 8636; 3 Program for Appropriate Technology in Health (PATH), Kyiv, Ukraine; 4 Implementation Research Division, The Aurum Institute, Johannesburg, South Africa; 5 KNCV Tuberculosis Plus, Dar es Salaam, United Republic of Tanzania; 6 Evidence and Impact, KNCV Tuberculosis Plus, The Hague, The Netherlands

**Keywords:** tuberculosis, digital adherence technology, DAT engagement, intervention fidelity, manual dosing, digital dosing

## Abstract

**Background:**

Digital adherence technologies (DATs) are promising tools for supporting tuberculosis (TB) treatment. DATs can serve as reminders for people with TB to take their medication and act as proxies for adherence monitoring. Strong engagement with DATs, from both the person with TB and health care provider (HCP) perspectives, is essential for ensuring intervention fidelity. The Adherence Support Coalition to End TB (ASCENT) project evaluated 2 types of DATs, pillboxes and medication labels (99DOTS), in cluster-randomized trials across 5 countries.

**Objective:**

This study aims to investigate participant and HCP engagement with DATs for TB treatment, stratified by DAT type and country.

**Methods:**

This study is a subanalysis of data generated through the ASCENT trials, which enrolled adults with drug-susceptible TB. A digital dose was defined as either a pillbox opening (for pillbox users) or a dosing confirmation SMS text message sent by the participant (for label users), both of which were recorded on the adherence platform. Descriptive analysis was used to provide an overview of dose-day outcomes. DAT engagement was assessed from both participant and HCP perspectives. To enhance participant engagement, we summarized the frequency of digital engagement overall and by treatment phase, as well as the frequency of consecutive days without engagement. For HCP engagement, we summarized the frequency of doses added manually, the number of days between the actual dose day and when a manual dose was added, and instances of consecutive manual dosing lasting more than 3 and more than 7 days, where doses were added more than 1 week after the dose day.

**Results:**

Of the 9511 participants included, 6719 (70.64%) were using the pillbox, 3544 (37.26%) were female, and the median age was 40 years. Across DAT types, there were 1,384,879 dose days, with 973,876 (70.32%) contributed by pillbox users. Of all dose days, 1,165,195 (84.14%) were recorded as digital, 156,664 (11.31%) as manual, 59,045 (4.26%) had no information, and 3975 (0.29%) were confirmed as missed. Digital dosing decreased slightly from the intensive to the continuation phase. The percentage of digital dose days was higher among pillbox users (851,496/973,876, 87.43%) compared with label users (313,699/411,003, 76.33%). Among label users, manual dosing was most common in the Philippines (37,919/171,786, 22.07%) and least common in Tanzania (11,108/76,231, 14.57%). Among pillbox users, manual dosing was most common in the Philippines (24,015/208,130, 11.54%) and Ukraine (13,209/111,901, 11.80%). Overall, 512 out of 2792 (18.34%) label users and 588 out of 6719 (8.75%) pillbox users experienced a run of more than 7 consecutive nondigital dose days that were resolved more than 1 week after the dose day. The highest occurrence was observed in the Philippines (368/1142, 32.22%, for label users and 224/1351, 16.58%, for pillbox users).

**Conclusions:**

There was considerable variation in DAT engagement across countries and DAT types, reflecting differences in how the intervention was implemented. Further refinement of the intervention and improvements in its delivery may be necessary to enhance outcomes.

## Introduction

Ending the tuberculosis (TB) epidemic is one of the globally agreed-upon development goals, with a target of reducing TB deaths by 90% by 2030 compared with the 2015 baseline [1]. However, poor treatment outcomes continue to pose significant challenges in high TB–burden settings [1]. Several interventions have been designed and implemented to improve treatment outcomes, including early diagnosis and treatment, counseling and psychosocial support, and managing comorbidities and coinfections [2]. These interventions are articulated within the broader directly observed therapy (DOTS) strategy, which emphasizes maximizing opportunities for direct observation of treatment. The standard treatment duration for drug-susceptible TB (DS-TB) is 6 months, comprising a 2-month intensive phase with 4 drugs followed by a 4-month continuation phase with 2 drugs. Treatment is taken daily during both phases, and direct in-person observation of dose intake remains the predominant recommendation worldwide [3].

With the increasing availability of technologies that facilitate remote treatment observation, alternatives to DOT, such as digital adherence technologies (DATs), have emerged as viable options. Several types of DATs have been used to support TB treatment, including SMS text message–based reminders, smart pillboxes, and video-supported therapy. A recent systematic review suggested that DAT use improves adherence in both DS-TB and drug-resistant TB treatment, although its effect on treatment outcomes remains mixed [4]. Scoping reviews of DAT implementations have documented intervention fidelity challenges, including technological and provider-facing issues, as well as complex cellular accessibility barriers [5,6]. While studies have summarized engagement with DATs from the perspective of individuals with TB, the perspective of health care providers (HCPs) has rarely been explored. A recent scoping review reported the weighted mean of digital engagement over dose days among people with TB as 87% (12 studies) for pillbox users and 61% (4 studies) for “99DOTS” users [6]. An analysis of 11 projects across 10 high TB-burden settings found that electronic doses added manually by HCPs (referred to as “manual doses”) accounted for 21.6% of reported doses in the DS-TB population [7].

Under the Adherence Support Coalition to End TB (ASCENT) project, we conducted pragmatic cluster-randomized trials in 5 countries to determine whether the use of DATs can improve treatment outcomes [8,9]. In these studies, eligible participants in the intervention arms received either pillboxes or medication labels (similar to “99DOTS” [10]), with the latter requiring participants to send a toll-free SMS text message each time treatment was taken. These DATs were integrated into a digital adherence platform, where participants’ digital engagement with the DATs (pillbox opened/SMS text messages sent) served as a proxy indicator of treatment adherence. Failure to engage with the DATs triggered a cascade of care actions, including automated SMS text message reminders, phone call reminders, and home visits, with escalating actions documented in response to higher levels of DAT nonengagement.

The objectives of this study were to describe the engagement of people with TB and HCPs with the DAT interventions, by country and DAT type. Our findings provide valuable insights into the implementation challenges of DAT interventions aimed at improving treatment adherence and outcomes for individuals with TB.

## Methods

### Study Design

We conducted pragmatic cluster-randomized trials in Ethiopia (PACTR2020087766949990), Ukraine, Tanzania, South Africa, and the Philippines (4 countries under ISRCTN17706019) to assess the effectiveness of DATs on TB treatment outcomes. The trial designs have been reported elsewhere [11,12]. Clusters, defined as health facilities (rayons in Ukraine), were purposively selected in consultation with the national TB programs based on previously notified TB cases, willingness and capacity to participate, and achieving a reasonable balance of urban and rural settings as well as large and small facilities. Clusters were selected from 5 oblasts in Ukraine, 4 regions in Tanzania, and 2 provinces in each of South Africa and the Philippines. Clusters were randomized (1:1:2 and 1:1:1 in Ethiopia) to receive either a smart pillbox, medication labels, or standard of care in all countries except Ukraine. As fixed-dose combinations were not available in Ukraine, the medication labels, which required fixed-dose combinations, were not used in the trial.

All adults with drug-sensitive TB starting the standard treatment regimen in facilities randomized to a DAT were offered enrollment into the study at treatment initiation by routine HCPs at the facility, following consent, using the DAT type allocated to their facility. Access to a mobile phone was a prerequisite for participation in the labels intervention. As such, participants in the medication labels arm without mobile phone access were offered the pillbox instead. Treatment supporter phone numbers could also be used in either DAT arm for participants without personal mobile phone access. Designated HCPs at each health facility responsible for TB care were trained in enrollment and in delivering the intervention, including the use of the digital adherence platform [13].

Participants using pillboxes were asked to keep their medication in the pillbox, which provided a daily audio/visual reminder at a predefined time based on participants’ preferred time to take their treatment. Box opening, a proxy for dose taken, was captured in real time on the cloud-based adherence platform, which could be viewed by HCPs at each facility. Participants using medication labels were asked to send a unique toll-free SMS text message code daily when taking their dose, with these data also captured on the adherence platform. A box opening or an SMS text message sent by a participant using labels was referred to as a digital dose. At a prearranged time, if no box-opening signal (for pillbox users) or SMS text message code (for label users) had been received, an automated SMS text message reminder to take and log the dose was sent from the adherence platform to participants with mobile phone access. No SMS text message reminders were used in Ukraine, based on a decision by the Ukrainian research team. HCPs reviewed dosing data on the adherence platform and employed strategies such as phone calls and home visits to promote medication adherence, based on a differentiated response algorithm. A dose not recorded digitally on the adherence platform but self-reported as taken by the participant following a phone call required manual adjustment of the recorded dose by the HCP on the adherence platform. Manual entries were expected to be completed promptly to meet adherence criteria and maintain the integrity of the treatment monitoring process, enabling timely intervention when needed.

Adults starting TB treatment in the standard-of-care arm received care according to their country’s guidelines, which could have involved facility-based or treatment-supporter–observed therapy, or self-administration.

In this analysis, we constructed a cohort of trial participants from the intervention arms of the 5 trials and used longitudinal data on their engagement with the DAT during their 6-month treatment period.

### Setting and Participants

We included adult participants enrolled in the trials who received either the pillbox or medication labels from June 1, 2021, to May 31, 2022. In South Africa, medication labels were discontinued early due to feasibility challenges; all participants enrolled after October 2021 were offered the pillbox. We excluded participants who switched from medication labels to pillboxes during their treatment. Participants were analyzed according to the DAT type received.

### Dosing Data

Data from the adherence platform were used to construct a profile of DAT engagement for each participant over their treatment period, from the DAT start to the earliest of (1) the treatment outcome date or (2) 168 days after treatment start (assuming 6 months of treatment). We excluded dose-day records with errors where either the date the dose was received was missing (10 records) or the date the dose was received preceded the dose day (36 records).

For each dose day, the adherence platform documented 1 of 4 mutually exclusive outcomes: digital dose, manual dose, dose missed, or no information. A digital dose-day confirmation refers to when the participant opens the pillbox/sends an SMS text message on the dose day. A manual dose refers to when the pillbox was not opened or an SMS text message was not sent on the dose day, but the HCP confirmed with the participant that the dose was taken and marked it as such on the platform. This confirmation of the dose being taken could occur on or after the actual dose day, with the date of confirmation recorded on the platform. A missed dose refers to instances when the pillbox was not opened or an SMS text message was not sent on the dose day, and the HCP later confirmed with the participant that the dose was not taken. Confirmation of both manual doses and doses not taken was based on participant self-report. “No information” for a dose day refers to instances when the pillbox was not opened or an SMS text message was not sent on the dose day, and no further information was available from the HCP regarding whether the dose was taken.

In real time, participants’ DAT engagement data were displayed in a calendar format on the platform, accessed by the HCPs (Figure S1 in Multimedia Appendix 1). Digital doses were shown in dark green. “No information” days, when the box was not opened or an SMS text message was not sent, were displayed in light red, starting from midnight the following day. If a “no information” dose day was later updated to a manual dose by the HCP, it was shown in light green. For confirmed missed doses, the dose day was changed to dark red. There were instances in the labels arm when participants correctly opened their pillbox or sent an SMS text message, but due to temporary connectivity issues, this information was not synced to the platform. As pillboxes maintain an on-device log of all openings, once connectivity was restored, the record of digital dosing would overwrite any interim activity, such as manual dosing entered by the HCP. This was not possible for medication labels, as the system did not support automatic resending of unreceived SMS text messages. There was no standardized approach to logging connectivity issues (except in Ethiopia), and when such issues did occur, they were generally facility-dependent.

The dataset used for the analysis was based on the last dose day recorded on November 15, 2022, and the data extract was dated December 7, 2022. A 3-week window was therefore allowed for any HCP activity, such as adding manual doses or overwriting data due to connectivity issues, to occur.

### Analyses

Descriptive analysis was used to provide an overview of dose-day outcomes. DAT engagement was categorized into 2 areas: participant engagement, represented by digital doses recorded on the platform; and HCP engagement, represented by dose days added manually. For participant engagement, we summarized the frequency of digital dosing overall, and separately for the intensive and continuation phases. For HCP engagement, we summarized the frequency of participants with manual dosing, as well as the timing of manual dose entries, defined as the number of days from the actual dose day to when a manual dose was added.

**Figure 1.**
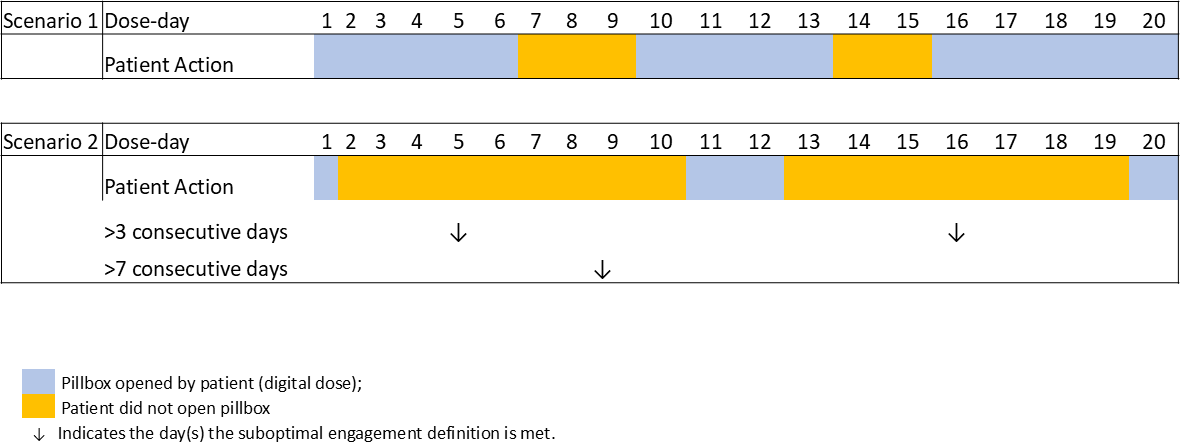
Illustration of 2 scenarios showing suboptimal engagement by person on tuberculosis treatment. Acceptable engagement is defined as ≤3 consecutive days with no digital confirmation. Suboptimal engagement is defined as (1) >3 consecutive dose days or (2) >7 consecutive dose days with no digital confirmation. Scenario 1: The patient had consecutive nondigital confirmation on days 7 to 9 (3 days; inclusive) and days 14 to 15 (2 days; inclusive); the suboptimal engagement condition of either >3 or >7 consecutive dose days is not met. Scenario 2: The patient had consecutive nondigital dose days on days 2 to 10 (9 days; inclusive) and days 13 to 19 (7 days; inclusive); the suboptimal engagement condition of (1) >3 dose days is met twice (on day 5 and day 16) and (2) >7 consecutive dose days is met once (on day 9).

We further defined scenarios reflecting “suboptimal engagement” with the DAT from both participant and HCP perspectives. From the participant’s perspective, suboptimal engagement was characterized by inadequate, timely interaction with the DAT, indicated by nondigital doses, defined as manual, missed, or no information dose days. We considered suboptimal, timely interaction to be present when there were more than 3 or more than 7 consecutive nondigital dose days. Figure 1 illustrates a case where suboptimal engagement criteria were met (scenario 2), whereas scenario 1 does not meet these criteria.

**Figure 2.**
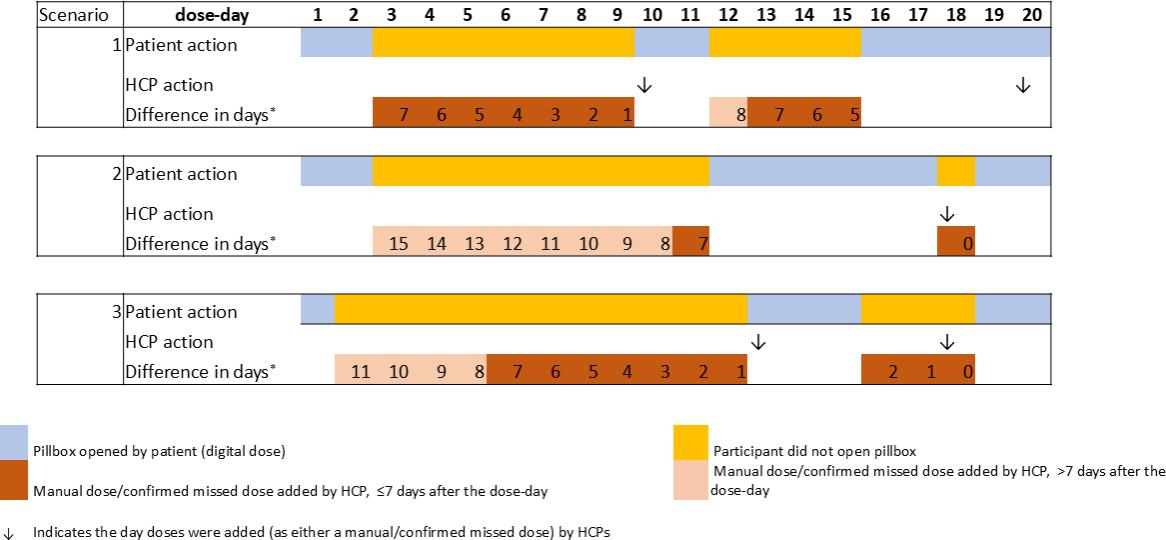
Illustration of 3 scenarios showing suboptimal engagement by health care professionals (HCPs). Suboptimal engagement is defined as (1) delayed action of manual/confirmed missed dose added by the HCP or no information about a dose and (2) this occurring for periods of consecutive days where non-digital dosing occurred. All scenarios show periods when the patient opened and did not open the pillbox. Scenario 1: HCP added manual/confirmed missed doses on days 10 and 20; of 11 doses added, 10 were within (≤) 7 days of the dose day; dose day 12 was added on day 20 (8 days late); the suboptimal engagement condition is not met. Scenario 2: HCP added manual/confirmed missed doses on day 18; of 10 doses added, 8 doses added were >7 days from the dose day; there is a run of 8 days where doses were added late (dose days 3-10; inclusive); the suboptimal engagement condition is met (consecutive dose days >3 and >7). Scenario 3: HCP added manual/confirmed missed doses on days 13 and 18; of 14 doses added, 4 were >7 days from the dose day; there is a run of 4 days where doses were added late (dose days 2-5; inclusive); the suboptimal engagement condition is met (consecutive dose days >3). *From actual dose day to when the dose was added (either as a manual or confirmed missed dose) by HCP.

From the HCP perspective, the scenario reflecting suboptimal engagement with the DAT was defined based on a combination of (1) delayed action, where manual or missed dose was added by the HCP, or there was no information about a dose; and (2) this occurring for periods of consecutive nondigital dose days. The first component was defined as a manual or missed dose added more than 7 days after the dose day, or the absence of any information more than 7 days after the dose day. A threshold of more than 1 week was chosen, as this was considered a sufficient time for the HCP to have successfully contacted the participant. For the second component, we considered periods of consecutive nondigital dose days, specifically more than 3 and more than 7 days, as instances when timely HCP interaction (ie, contact with the participant) would have been expected. See Figure 2 for illustrative scenarios. Scenarios 2 and 3 meet the criteria for suboptimal engagement, whereas scenario 1 does not. Analyses, stratified by country and DAT type, were conducted using Stata version 17 (StataCorp).

### Ethical Considerations

Written informed consent was obtained from all participants initiating a DAT in the ASCENT trials. The main trials were approved by the World Health Organization (WHO) Ethical Review Committee, Switzerland; Institutional Review Boards in Ethiopia (Addis Ababa City Administration Health Bureau Public Emergency and Health Research Directorate Institutional Review Board, and Oromia Regional Health Bureau Public Emergency and Health Research Directorate Institutional Review Board); the Ethics Committee for the Philippines (Single Joint Research Ethics Board), South Africa (University of the Witwatersrand Human Research Ethics Committee), and Tanzania (Tanzania Medical Research Coordinating Committee at the National Institute for Medical Research, Dar es Salaam); and the London School of Hygiene & Tropical Medicine Ethics Committee, United Kingdom. The analyses presented here are covered by the original ethics approvals and did not require additional consent. All data were deidentified and contained no personal identifiers. Trial participants did not receive any compensation for their participation.

## Results

### Participant Characteristics

Overall, 9511 participants from 163 health facilities/rayons contributed to the analysis, of whom 6719 (70.64%) were using the pillbox: 1243 out of 2268 (54.81%) in Ethiopia, 1698 out of 1759 (96.53%) in South Africa, 1611 out of 2185 (73.73%) in Tanzania, 1351 out of 2493 (54.19%) in the Philippines, and 816 out of 816 (100%) in Ukraine. In total, 3544 (37.26%) were female: 936 out of 2268 (41.27%) in Ethiopia, 676 out of 1749 (38.65%) in South Africa, 824 out of 2185 (37.71%) in Tanzania, 845 out of 2493 (33.89%) in the Philippines, and 263 out of 816 (32.23%) in Ukraine. The median age was 40 years (29 years in Ethiopia, 41 years in South Africa, 45 years in Tanzania, 45 years in the Philippines, and 44 years in Ukraine; see Table 1).

**Table 1.** Baseline characteristics of study participants by country of enrollment and DAT^a^ received.

Country and DAT type	Number of participants (number of facilities), n	Male, n (%)	Female, n (%)	Transgender, n (%)	Age (years), median (Q1^b^-Q3)^c^
Ethiopia					
Labels	1025 (26)	607 (59.22)	418 (40.78)	0 (0)	28 (23‐39)
Pillbox	1243 (49)	725 (58.33)	518 (41.67)	0 (0)	30 (24‐41)
South Africa					
Labels	51 (9)	35 (68.63)	16 (31.37)	0 (0)	37 (31‐44)
Pillbox	1698 (30)	1038 (61.13)	660 (38.87)	0 (0)	41 (33‐51)
Tanzania					
Labels	574 (16)	369 (64.29)	205 (35.71)	0 (0)	41 (30‐54)
Pillbox	1611 (36)	992 (61.58)	619 (38.42)	0 (0)	46 (34‐60)
The Philippines					
Labels	1142 (16)	745 (65.24)	395 (34.59)	2 (0.18)	44 (29‐55)
Pillbox	1351 (27)	901 (66.69)	450 (33.31)	0 (0)	46 (32‐59)
Ukraine					
Pillbox	816 (12)	553 (67.77)	263 (32.23)	0 (0)	44 (35‐54)
Overall	9511 (163)	5965 (62.72)	3544 (37.26)	2 (0.02)	40 (29‐53)

a DAT: digital adherence technology.

b Q1: lower quartile.

c Q3: upper quartile.

### Description of Dosing Type

During the follow-up period, a total of 1,384,879 dose days from 9511 participants—across both DAT types—contributed to the analysis. The median number of dose days per participant was 168 days. Of all dose days, 973,876 out of 1,384,879 (70.32%) were among participants using pillboxes. Overall, 1,165,195 out of 1,384,879 (84.14%) dose days were recorded digitally, 156,664 out of 1,384,879 (11.31%) manually, 59,045 out of 1,384,879 (4.26%) had no information, and fewer than 3975 out of 1,384,879 (0.29%) were confirmed as missed. A higher percentage of dose days were recorded digitally among pillbox users compared with label users (851,496/973,876, 87.43% vs 313,699/411,003, 76.33%), with patterns varying by country (Table 2). Manual dosing for labels was most common in the Philippines (37,919/171,786, 22.07%, dose days) and least common in Tanzania (11,108/76,231, 14.57%, dose days). Among pillbox users, manual dose days were most common in the Philippines and Ukraine (24,015/208,130, 11.54%, and 13,209/111,901, 11.80%, dose days, respectively) and least common in South Africa (9075/235,417, 3.85%, dose days). No information for the dose day was most common among participants using labels (1190/6154, 19.34%, dose days) and pillboxes (18,711/235,417, 7.95%, dose days) in South Africa. Manual doses were added a median of 4 (lower quartile-upper quartile 1‐16) days after the dose day for participants using labels, and 6 (lower quartile-upper quartile 2‐21) days for those using pillboxes. The median time from the dose day to manual dose being added was shortest for participants in Ethiopia (2 days for labels and 3 days for pillboxes) and longest for participants in the Philippines (9 days for labels and 12 days for pillboxes). The Philippines had the highest percentage of manual doses added more than 7 days after the dose day (20,831/37,919, 54.94%, for labels and 14,708/24,015, 61.25%, for pillboxes), followed by South Africa (541/1149, 47.08%, for labels and 3966/9075, 43.70%, for pillboxes) and Ukraine (6858/13,209, 51.92%, for pillboxes). Ethiopia had the lowest proportions (6016/28,783, 20.90%, for labels and 5075/16,845, 30.13%, for pillboxes; see Table 2).

Digital dosing decreased slightly from the intensive compared with the continuation phase (see Figure S2 in Multimedia Appendix 1). The median number of days to add manual doses increased slightly during the continuation phase compared with the intensive phase for label users in the Philippines (from 8 to 10 days) and for pillbox users in Ukraine (from 7 to 10 days). For other countries and DAT types, the median number of days remained similar (see Table S1 in Multimedia Appendix 1).

**Table 2.** Summary of dose-day types and time to manual dose entry over the 6-month treatment period, by country of enrollment and digital adherence technology received.

Country	Labels	Pillbox	
Ethiopia			
Recording of dose, day level			
Digital, n (%)	126,718 (80.80)	177,599 (90.45)	
Manual, n (%)	28,783 (18.35)	16,845 (8.58)	
Missed, n (%)	309 (0.20)	144 (0.07)	
No information, n (%)	1022 (0.65)	1764 (0.90)	
Total, n	156,832	196,352	
Days from the dose day to when the manual dose was added to the platform			
Median days (Q1^a^-Q3^b^)	2 (1-5)	3 (1-10)	
Same day, n (%)	5665 (19.68)	3559 (21.13)	
1‐7 days, n (%)	17,102 (59.42)	8211 (48.74)	
>7 days, n (%)	6016 (20.90)	5075 (30.13)	
South Africa			
Recording of dose, day level			
Digital, n (%)	3815 (61.99)	207,569 (88.17)	
Manual, n (%)	1149 (18.67)	9075 (3.85)	
Missed, n (%)	0 (0)	62 (0.03)	
No information, n (%)	1190 (19.34)	18,711 (7.95)	
Total, n	6154	235,417	
Days from the dose day to when the manual dose was added to the platform			
Median days (Q1-Q3)	6 (2-20)	6 (2-19)	
Same day, n (%)	149 (12.97)	727 (8.01)	
1‐7 days, n (%)	459 (39.95)	4382 (48.29)	
>7 days, n (%)	541 (47.08)	3966 (43.70)	
Tanzania			
Recording of dose, day level			
Digital, n (%)	63,862 (83.77)	202,382 (91.13)	
Manual, n (%)	11,108 (14.57)	14,561 (6.56)	
Missed, n (%)	213 (0.28)	657 (0.30)	
No information, n (%)	1048 (1.37)	4476 (2.02)	
Total, n	76,231	222,076	
Days from the dose day to when the manual dose was added to the platform			
Median days (Q1-Q3)	3 (1-8)	4 (2-10)	
Same day, n (%)	551 (4.96)	1174 (8.06)	
1‐7 days, n (%)	7651 (68.88)	8764 (60.19)	
>7 days, n (%)	2906 (26.16)	4623 (31.75)	
The Philippines			
Recording of dose, day level			
Digital, n (%)	119,304 (69.45)	172,234 (82.75)	
Manual, n (%)	37,919 (22.07)	24,015 (11.54)	
Missed, n (%)	1756 (1.02)	384 (0.18)	
No information, n (%)	12,807 (7.46)	11,497 (5.52)	
Total, n	171,786	208,130	
Days from the dose day to when the manual dose was added to the platform			
Median days (Q1-Q3)	9 (3-24)	12 (4‐17)	
Same day, n (%)	2381 (6.28)	932 (3.88)	
1‐7 days, n (%)	14,707 (38.79)	8375 (34.7)	
>7 days, n (%)	20,831 (55)	14,708 (61.25)	
Ukraine			
Recording of dose, day level			
Digital, n (%)	N/A^c^	91,712 (81.96)	
Manual, n (%)	N/A	13,209 (11.80)	
Missed, n (%)	N/A	450 (0.40)	
No information, n (%)	N/A	6530 (5.84)	
Total, n	N/A	111,901	
Days from the dose day to when the manual dose was added to the platform			
Median days (Q1-Q3)	N/A	9 (3-24)	
Same day, n (%)	N/A	599 (4.53)	
1‐7 days, n (%)	N/A	5752 (43.55)	
>7 days, n (%)	N/A	6858 (51.92)	
Overall			
Recording of dose, day level			
Digital, n (%)	313,699 (76.33)	851,496 (87.43)	
Manual, n (%)	78,959 (19.21)	77,705 (7.98)	
Missed, n (%)	2278 (0.55)	1697 (0.17)	
No information, n (%)	16,067 (3.91)	42,978 (4.41)	
Total, n	411,003	973,876	
Days from the dose day to when the manual dose was added to the platform			
Median days (Q1-Q3)	4 (1-16)	6 (2-21)	
Same day, n (%)	8746 (11.08)	6991 (9.00)	
1‐7 days, n (%)	39,919 (50.56)	35,484 (45.67)	
>7 days, n (%)	30,294 (38.37)	35,230 (45.34)	

a Q1: lower quartile.

b Q3: upper quartile.

c N/A: not applicable.

### Participant and HCP Suboptimal DAT Engagement

**Figure 3.**
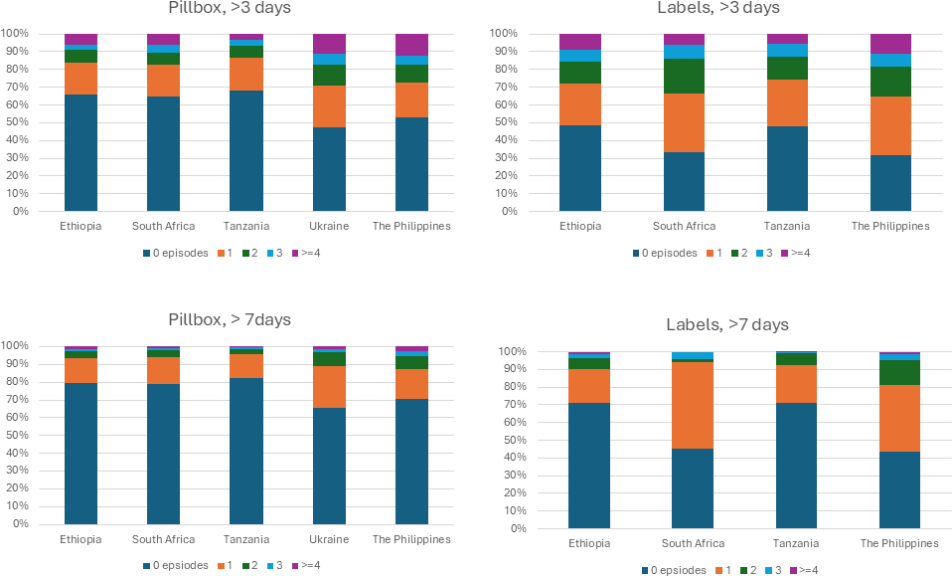
Bar charts showing the distribution of the total number of participant-level episodes (grouped) of consecutive nondigital dose days lasting >3 and >7 days over the 6-month treatment period, stratified by country of enrollment and type of digital adherence technology received. Top panels: summary at the participant level of the number of episodes (grouped into 0, 1, 2, 3, and ≥4 episodes) where >3 consecutive non-digital dose-days were observed for pillbox (left) and labels (right), by country. Lower panels: summary at the participant level of the episodes (grouped into 0, 1, 2, 3, and ≥4 episodes) where >7 consecutive non-digital dose-days were observed for pillbox (left) and labels (right), by country.

Participant suboptimal engagement analysis is summarized in Figure 3. From the participant’s perspective, the Philippines had the highest number of episodes of more than 3 consecutive nondigital dose days for both pillboxes and labels, while Tanzania and Ethiopia had the lowest frequency. For episodes of more than 7 consecutive nondigital dose days, the Philippines again showed the highest frequency for both DAT types, with the lowest frequency observed in Ethiopia and South Africa.

Suboptimal engagement by the HCP is summarized in Table 3. Of the 2792 participants using labels, 820 (29.37%) and 512 (18.34%) met the criteria for suboptimal engagement of more than 3 days and more than 7 days, respectively, at least once. The Philippines had the highest percentage of HCP suboptimal engagement, with 501 out of 1142 (43.87%) and 368 out of 1142 (32.22%) participants experiencing suboptimal engagement, at least once, of more than 3 days and more than 7 days, respectively. South Africa ranked second, with 19 of 51 (37%) and 15 of 51 (29%) for suboptimal engagement of more than 3 days and more than 7 days, respectively. Combining across the 4 countries, 909 out of 1698 (53.53%) episodes lasting more than 3 days also met the criterion for more than 7 days.

**Table 3.** Suboptimal health care provider engagement with the digital adherence technology over the 6-month treatment period, by country of enrollment and type of digital adherence technology received.^a^

DAT type, country (number of participants), and consecutive nondigital runs^b^			Total number of episodes, n	Number of participants with at least one episode, n (%)	Summary of number of episodes, participant-level, median (Q1^c^-Q3^d^)
Labels					
Ethiopia (n=1025)					
>3 days			368	208 (20.3)	1 (1-2)
>7 days			183	103 (10.0)	1 (1-2)
South Africa (n=51)					
>3 days			39	19 (37.3)	1 (1-3)
>7 days			30	15 (29.4)	2 (1-2)
Tanzania (n=574)					
>3 days			135	92 (16.0)	1 (1-2)
>7 days			31	26 (4.5)	1 (1-1)
The Philippines (n=1142)					
>3 days			1156	501 (43.9)	2 (1-3)
>7 days			665	368 (32.2)	1 (1-2)
Pillboxes					
Ethiopia (n=1243)					
>3 days			349	185 (14.9)	1 (1-2)
>7 days			157	98 (7.9)	1 (1-2)
South Africa (n=1698)					
>3 days			241	138 (8.2)	1 (1-2)
>7 days			114	70 (4.1)	1 (1-2)
Tanzania (n=1611)					
>3 days			284	175 (10.9)	1 (1-2)
>7 days			103	79 (4.9)	1 (1-1)
The Philippines (n=1351)					
>3 days			825	347 (25.7)	2 (1-3)
>7 days			407	224 (16.6)	1 (1-2)
Ukraine (n=816)					
>3 days			388	217 (26.6)	1 (1-2)
>7 days			158	117 (14.3)	1 (1-2)

a Suboptimal engagement was defined as (1) delayed action by the provider—either manually adding a missed dose or providing no dose information—more than 7 days after the dose day, and (2) this delay occurring during runs of more than 3 or more than 7 consecutive nondigital dose days.

b For doses added more than 7 days after the dose day.

c Q1: lower quartile.

d Q3: upper quartile.

Among participants using the pillbox (n=6719), 1062 (15.81%) and 588 (8.75%) met the criteria for suboptimal engagement of more than 3 days and more than 7 days, respectively, at least once. Ukraine had the highest proportion of participants with more than 3 days (217/816, 26.6%) and more than 7 days (117/816, 14.3%) of suboptimal engagement, followed by the Philippines (347/1351, 25.7% and 224/1351, 16.6%, respectively). Combining across the 5 countries, of the 2087 episodes lasting more than 3 days, 939 (45.0%) also met the definition for more than 7 days of suboptimal engagement. Typically, participants experienced a median of 1 episode of suboptimal engagement, except in the Philippines and South Africa. In the Philippines, the median number of episodes lasting more than 3 days was 2 for both DATs. In South Africa, the median number of episodes lasting more than 7 days was 2 for the label DAT.

## Discussion

### Principal Findings

This study sought to understand the complex interactions between adults on DS-TB treatment, HCPs, and the DAT intervention, using daily DAT engagement data from 2 DAT types across 5 countries. We found a higher percentage of dose days recorded as digital among pillbox users compared with label users, with digital dosing lowest among pillbox users in Ukraine and the Philippines. The time taken by HCPs to add manual doses varied across countries: the Philippines had the longest intervals for both DAT types, followed by Ukraine and South Africa. Combining across DATs, 34,537 out of 45,628 (75.69%) manual doses in Ethiopia and 18,140 out of 25,669 (70.67%) in Tanzania were added within a 7-day time frame. There was also a lack of dose-taking information recorded on the adherence platform, most frequently observed in South Africa, reflecting another marker of poor HCP engagement with the intervention. Finally, our marker of suboptimal DAT engagement by HCPs showed variation by DAT type and country. Across countries, a range of label users (92/574, 16.0%, to 501/1142, 43.9%) and pillbox users (138/1698, 8.13%, to 217/816, 26.6%) experienced at least one run of nondigital dosing lasting more than 3 days, which was either resolved more than 7 days after the dose day or not resolved at all (no information). The Philippines had the highest percentage of participants experiencing this issue for labels (501/1142, 43.87%) and, along with Ukraine, the highest for pillboxes (347/1351, 25.7%, and 217/816, 26.6%, respectively).

We observed differences in our indicators by country, which may be explained by variations in participants, health care systems, and external factors and are consistent with our process evaluation [13]. The findings from the Philippines stand out, reflecting a pattern of lower engagement with the DATs by both participants and HCPs. Higher levels of manual dosing and more frequent episodes of consecutive nondigital dosing suggest lower participant engagement in this setting. This is further supported by findings from the ASCENT acceptability substudy, where participants in the Philippines reported lower mean scores for Capability, Opportunity, and Motivation, key domains of the Theoretical Domains Framework used to assess acceptability, compared with participants in other countries [14]. Participants also reported lower HCP engagement in the acceptability substudy, as indicated by a lower percentage reporting that HCPs showed them their adherence data during routine clinic visits. In the Philippines, intervention delivery was affected by the reassignment of TB focal staff to COVID-19 vaccination programs in 2021. This led to a decline in participant enrollment during that period and likely posed challenges in delivering the DAT intervention to those already enrolled. In a qualitative study involving people with TB and HCPs participating in the Philippines trial, HCPs cited insufficient training and lack of refresher training at the program design level, as well as power issues at the community level, as barriers to DAT implementation [15]. In South Africa, missing dose information on the platform may have been due to difficulties in contacting participants, such as changes in phone numbers, loss of phones, or provision of incorrect contact details, hindering HCPs’ ability to confirm doses. Additionally, some participants were lost to follow-up due to mobility-related issues, such as relocating for work, which resulted in unsuccessful home visits for dose confirmation. During the study period, frequent power outages in South Africa may have contributed to suboptimal HCP engagement with the intervention. In Ukraine, the study coincided with disruptions caused by the military invasion by Russia, which likely affected the timeliness of HCP contact with participants following missed doses recorded on the platform. Regional differences in dosing patterns were observed within Ukraine; Mykolaivska and Donetska oblasts, occupied by Russia early in the invasion, had higher levels of manual dosing added late compared with other regions [16]. Furthermore, in Ukraine, the DAT intervention was implemented by HCPs within the context of staff reductions in TB care delivery, a challenge not reported in other countries. By the midpoint of trial implementation, fewer HCPs were providing DAT support than had been trained at the start of the trial, which may have contributed to suboptimal engagement with the DAT.

In the acceptability substudy, regular access to a mobile phone was identified as an important aspect of the intervention from both participant and HCP perspectives. Shared phone ownership may hinder timely dose taking in response to automated SMS text message reminders in either arm, as well as limit HCPs’ ability to successfully contact participants following nonengagement. Ownership of a nonshared phone was lowest among participants in the Philippines compared with other countries, potentially contributing to suboptimal engagement from both participant and HCP perspectives [14]. Disparities in phone ownership are well-known challenges in implementing digitally mediated interventions [17,18].

Poor connectivity poses a significant barrier to optimal DAT engagement, particularly affecting the label intervention. Unlike the pillbox intervention, where box openings are transmitted once connectivity is restored, the label intervention cannot “correct” for periods of nonconnectivity. This limitation may have contributed to lower recorded digital doses for labels compared with pillboxes, as missed or delayed transmissions could not be retrospectively updated. Consequently, participants using labels may appear less engaged, even if their actual adherence was higher than reflected in the data. This discrepancy can also affect health care workers’ engagement with DATs, as they may become less motivated to rely on or use the technology if they perceive it as unreliable [5,19,20]. Addressing connectivity issues and incorporating features to mitigate their impact are crucial for ensuring accurate data capture and improving overall engagement from both participants and health care workers.

### Comparison With Prior Work

Various levels of participant engagement have been documented across different DAT types in various settings. A recent scoping review reported lower participant engagement with 99DOTS (similar to our labels interventions) compared with pillboxes [6]. Furthermore, factors such as suboptimal DAT functionality, complex cellular acceptability, and less accurate adherence data may have contributed to these discrepancies [5]. This could be partly explained by differences in ease of use: pillbox users received an optional daily audio reminder, as well as an automated SMS text message reminder if the system did not receive a pillbox opening signal or SMS text message dose confirmation from the participant within a set time. Ready access to a phone among label users may have been a challenge for documenting dose-taking. In the 99DOTS stepped-wedge trial in Uganda, the median percentage of digital dosing was 58.4%, peaking in month 1 at 71.4% and declining to 46.4% by month 6. When manual dosing was included, the median percentage exceeded 99.4% [21]. Digital dosing among label users in the ASCENT trials was generally higher compared with this study. The percentage of digital dosing recorded in pillbox intervention arms in other trials ranges from 84.2% to 88%, aligning with the ASCENT trial data [22-24].

Few studies, however, have summarized digital/nondigital dosing patterns from both participant and HCP perspectives. A meta-analysis of data from 11 projects provided a valuable overview of DAT engagement patterns over the treatment period, but did not explore the nuances of manual dosing frequency and timing. This may be due to differences in how manual dosing was defined across studies, as well as limitations in data capture that prevented clear distinctions between digital and manual dosing [7]. The significance of consecutive nondigital doses, particularly when frequent and prolonged, may indicate participant nonengagement with the DAT. In such cases, manual doses added late by HCPs could also reflect poor HCP engagement. High fidelity in intervention delivery by HCPs is essential for the success of DATs. A multicomponent intervention conducted in Tibet among 276 individuals starting TB treatment, which included pillboxes and a switch to video-supported therapy for those with adherence challenges, reported high fidelity to the transition to video-supported therapy, with at least 89% maintained over the treatment period [25]. In our trials, the calendar format on the adherence platform provided HCPs with a clear visualization of patients’ dose taking, intended to support timely action. However, concerns have been raised about the use of this format, particularly in busy health facilities. There may be a temptation for HCPs to manually record doses in the digital calendar without clear confirmation from the participant, similar to critiques of adherence documentation in TB register calendars used in standard care.

### Limitations

Limitations of our study include the fact that DAT engagement reflects only dosing as recorded on the adherence platform; therefore, digital dosing should not be directly equated with actual pill consumption. What was recorded were proxies (either via SMS text messages or pillbox signals) of medication intake. Manual doses added late may have resulted from HCPs being unable to successfully contact participants, rather than from delayed action or lack of attempt. However, we were unable to distinguish between these possibilities. Hence, caution should be exercised when interpreting the findings. Furthermore, the external validity is limited, as the sampled participants and collected data were specific to the ASCENT trial. Nonetheless, the strengths of our study are its coverage of 5 countries, each with distinct epidemiological, socioeconomic, geographical, infrastructural, and health system contexts. Data were generated by routine staff delivering the intervention across 163 health facilities/rayons and included 9511 participants.

### Conclusions

This study highlights the role of DATs in TB treatment, focusing on how adults with DS-TB and HCPs interact with the technology. We observed considerable variation in DAT engagement across countries and DAT types, reflecting differences in how the intervention was implemented. These findings underscore the need for further research to assess whether variations in DAT engagement affect the overall effectiveness of these interventions. Such research could help inform improvements in DAT design and implementation to foster higher engagement among people with TB and HCPs, thereby potentially enhancing intervention effectiveness.

## Supplementary material

10.2196/62881 Multimedia Appendix 1 Additional analysis.

10.2196/62881 Checklist 1 STROBE checklist.

## Abbreviations

ASCENT: Adherence Support Coalition to End TB
DAT: digital adherence technology
DOTS: directly observed therapy
DS-TB: drug-susceptible tuberculosis
HCP: health care provider
TB: tuberculosis
WHO: World Health Organization
