# Supplementary material for: Engagement With Digital Adherence Technologies as Measures of Intervention Fidelity Among Adults With Drug-Susceptible Tuberculosis and Health Care Providers: Descriptive Analysis Using Data From Cluster-Randomized Trials in Five Countries
Source: JMIR Public Health Surveill. 2025 Jul 28;11:e62881. doi: 10.2196/62881 (PMC12303541; doi:10.2196/62881)
Supplement: Multimedia Appendix 1 [file publichealth-v11-e62881-s001.docx]

**Supplement**

**Figure S1: Adherence calendar illustrating the ‘dose-taken’ status of the participant on each day, indicated by the colour for that date.**


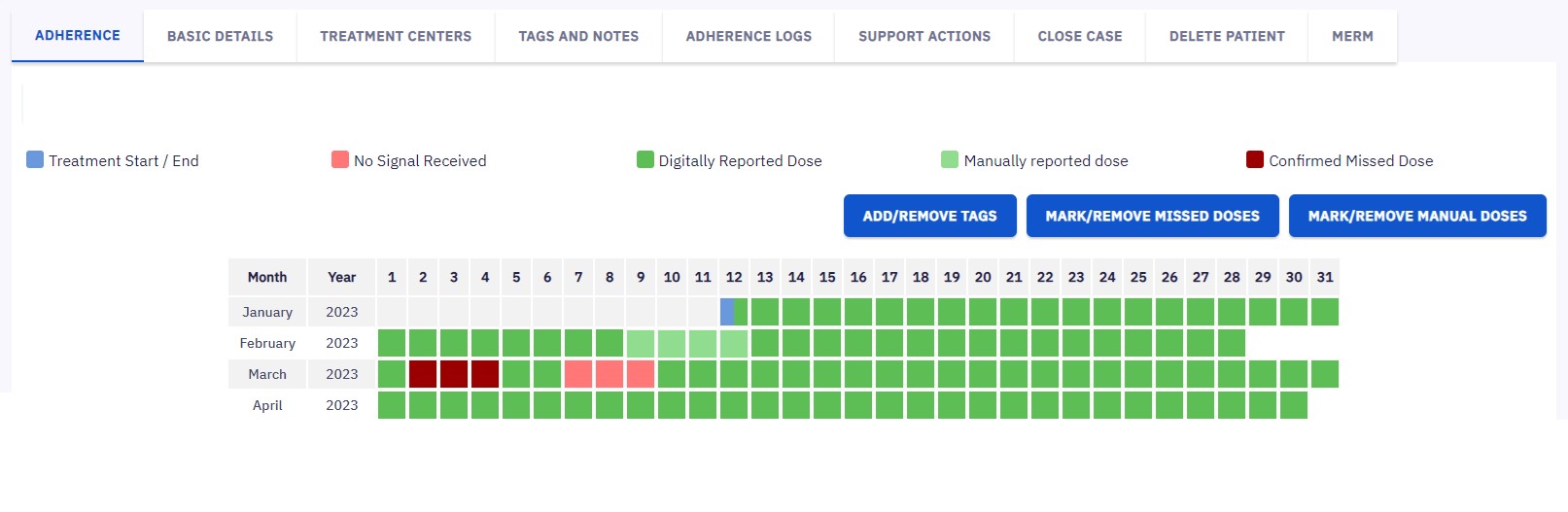


Footnote: Digital doses are shown in dark green. No signal received show days without information (when the box was not opened, or an SMS was not sent) are displayed in light red. If a no-information dose-day was changed to a manual dose by the HCP, it appears in light green. Confirmed missed doses are marked in dark red.

**Figure S2: dose day type by country, DAT type received and treatment phase**

Footnote: int intensive phase; cont continuation phase

Digital - participant opens the pillbox/sends an SMS on the dose-day; manual - pillbox was not opened or SMS was not sent on the dose-day, but the HCP confirmed with the participant the dose was taken and marked as such on the platform; missed - pillbox was not opened/SMS was not sent on the dose day, and the HCP confirmed with the participant that the dose was not taken; no info - pillbox was not opened/SMS was not sent on the dose-day, and there was no further information from the HCP as to whether the dose was taken or not.

**Table S1: summary of days from dose-day to when manual dose was added stratified by treatment phase (intensive vs continuation), DAT type and country**

|  | **Intensive phase** | | | **Continuation phase** | | |
| --- | --- | --- | --- | --- | --- | --- |
| **Labels** | **median** | **IQR** | **n** | **median** | **IQR** | **n** |
| Ethiopia | 1 | 1,5 | 8,265 | 2 | 1,5 | 20,518 |
| South Africa | 9 | 2,21 | 409 | 6 | 2,17 | 740 |
| Tanzania | 3 | 1,7 | 3,193 | 3 | 1,8 | 7,915 |
| The Philippines | 8 | 3,23 | 12,032 | 10 | 3,24 | 25,887 |
| **Pillbox** | **median** | **IQR** | **n** | **median** | **IQR** | **n** |
| Ethiopia | 3 | 1,11 | 4,184 | 3 | 1,10 | 12,661 |
| South Africa | 5 | 2,16 | 2,966 | 6 | 2,20 | 6,109 |
| Tanzania | 3 | 1,10 | 4,674 | 4 | 2,11 | 9,887 |
| The Philippines | 11 | 4,28 | 6,913 | 12 | 4,27 | 17,102 |
| Ukraine | 7 | 1,23 | 3,841 | 10 | 1,32 | 9,358 |

IQR interquartile range
